# Prevalence of specific immunoglobulin E and G against *Aspergillus fumigatus* in patients with asthma

**DOI:** 10.18502/cmm.4.4.380

**Published:** 2018-12

**Authors:** Newsha Hedayati, Vida Mortezaee, Seyed Alireza Mahdaviani, Maryam Sadat Mirenayat, Maryam Hassanzad, Mihan Pourabdollah, Jalal Heshmatnia, Atefeh Fakharian, Guitti Pourdolat, Somayeh Sharifynia, Mahshid Vakili, Mahdi Abastabar, Iman Haghani, Masoud Aliyali, Hossein Asgarian-Omran, Mohammad T. Hedayati

**Affiliations:** 1 Student Research Committee, Mazandaran University of Medical Sciences, Sari, Iran; 2 Student Research Committee, Invasive Fungi Research Center, Mazandaran University of Medical Sciences, Sari, Iran; 3 Pediatric Respiratory Diseases Research Center, National Research Institute of Tuberculosis and Lung Diseases, Shahid Beheshti University of Medical Sciences, Tehran, Iran; 4 Chronic Respiratory Diseases Research Center, National Research Institute of Tuberculosis and Lung Diseases, Shahid Beheshti University of Medical Sciences, Tehran, Iran; 5 Clinical Tuberculosis and Epidemiology Research Center, National Research Institute of Tuberculosis and Lung Diseases, Shahid Beheshti University of Medical Sciences, Tehran, Iran; 6 Invasive Fungi Research Centre, Mazandaran University of Medical Sciences, Sari, Iran; 7 Department of Medical Mycology, Mazandaran University of Medical Sciences, Sari, Iran; 8 Pulmonary and Critical Care Division, School of Medicine, Mazandaran University of Medical Sciences, Sari, Iran; 9 Department of Immunology, School of Medicine, Mazandaran University of Medical Sciences, Sari, Iran

**Keywords:** Aspergillus fumigatus, Asthmatic patients, Specific IgE, Specific IgG

## Abstract

**Background and Purpose:**

*Aspergillus fumigatus *as a ubiquitous fungus can be found in the respiratory tract of the asthmatic and healthy people. The inhalation of *Aspergillus* spores leads to an immune response in individuals with asthma and results in the aggravation of the clinical symptoms. The present study aimed to investigate the prevalence of specific immunoglobulin E and G** (**IgE and IgG) against *A.fumigatus *in asthmatic patients.

**Materials and Methods:**

This study was conducted on 200 consecutive patients with moderate to severe asthma referring to Masih Daneshvari hospital Tehran, Iran, from January 2016 to February 2018. Skin prick test (SPT) was performed in all subjects with *Aspergillus* allergens. Moreover, all patients underwent specific IgE testing for *Aspergillus* using Hycor method. Enzyme immune assay was applied to measure total IgE and *Aspergillus-*specific IgG.

**Results:**

According to the results, the mean age of the patients was 45.8 years (age range: 18-78 years). The mean levels of total IgE and *Aspergillus *specific IgE in asthmatic patients were obtained as 316.3 (range: 6-1300 IU/ml) and 1.5 (range: 0.1-61.3 IU/ml), respectively. Out of 200 patients, 27 (13.5%), 65 (32.5%), 22 (11.0%), and 86 (43.0%) cases had positive *Aspergillus* SPT, total IgE of > 417 IU/ml, *Aspergillus***-**specific IgE, and IgG, respectively. The level of these variables in patients with severe asthma were 16 (16.5%), 36 (37.1%), 15 (15.5%), and 46 (47.4%), respectively.

**Conclusion:**

As the findings indicated, reactivity to *Aspergillus* is a remarkable phenomenon in asthmatic patients. It is also emphasised that the climatic condition may affect the positive rate of hypersensitivity to *Aspergillus*.

## Introduction

Bronchial asthma as a global public health problem is a complex inflammatory and heterogeneous disease of chronic inflammation in airways with significant morbidity and mortality. Asthma characterized by clinical symptoms, airflow limitation, airway hyper-responsiveness, chest tightness, recurrent episodes of breathlessness, dyspnoea, cough, and wheeze (1-4). Furthermore, 5-10% of all asthmatic patients suffer from poorly controlled and severe asthma which limits daily activities and may result in death (1-3). Environmental factors, such as animal dander, house dust mite, and exposure to airborne fungal spores, are heavily involved in the development of asthma and deterioration of clinical signs (3, 4). 

Moreover, fungal allergens can aggravate asthma due to atopic condition which is a hallmark of asthma (5). In severe manifestations of asthma, fungal colonization and sensitivity to fungal allergens can lead to asthma exacerbation (4, 6). 

Denning et al. proposed the term "severe asthma with fungal sensitization" (SAFS) to describe the severe asthma in patients who are sensitive to fungi without any evidence of allergic bronchopulmonary aspergillosis (ABPA) diagnosis and lack of response to antifungal therapy (7). It is estimated that approximately 20-25% of people with stable severe asthma are affected by SAFS (6, 8). Sensitization to different fungal genera is reported in asthmatic patients; however, *Aspergillus* sensitivity was more frequent in SAFS and asthma associated to fungal sensitization (AAFS) (9). 

*Aspergillus fumigatus *as a ubiquitous fungus can be found in the respiratory tract of the asthmatic and healthy people (10, 11). The inhalation of *Aspergillus* spores leads to an immune response in individuals. Moreover, it stimulates inflammatory responses by T helper 2, whereby IgE and IgG antibodies act against *A.fumigatus *(3). *A. fumigatus* may sensitize the patients to asthma, leading to IgE-mediated hypersensitivity and ABPA with variable prevalence (12). The evaluation of serum specific IgE and IgG against *A.fumigatus *is considered as the main criteria for the diagnosis of allergic diseases due to *Aspergillus*. Therefore, the aim of the present study was to determine the prevalence of specific IgE and IgG against *A.fumigatus *in patients with moderate and severe asthma.

## Materials and Methods

This study was conducted on a total of 200 consecutive patients referring to Masih Daneshvari hospital, Tehran, Iran from January 2016 to February 2018. The subjects with the age range of 18-78 years were diagnosed with moderate to severe asthma based on the Global Initiative for Asthma guidelines (2015, https://ginasthma.org). In addition, all participants were asked to fill demographic forms.

The exclusion criteria were: 1) pregnancy, 2) other widespread lung diseases (e.g., tuberculosis), 3) cystic fibrosis, 4) mild asthma, and 5) malignancy. Spirometry was performed according to European Respiratory Society guidelines (13). Furthermore, all subjects underwent high resolution computed tomography. The study was approved by the Ethics Committee of Mazandaran University of Medical Sciences, Sari, Iran, (IR.MAZUMS.REC.95.2412) and the written consent was taken from all included participants.

***Aspergillus Skin Prick Test***

Skin prick testing was performed in all subjects with *Aspergillus* allergens (Alk-Abello, Lincoln Diagnostics, Dallas, Tx, USA). Histamine and normal saline were used as positive and negative controls, respectively. A positive *Aspergillus* skin prick test (AST) reaction in negative control was defined as the appearance of a wheal greater than 3 mm 15 min after exposure.

***Aspergillus-specific IgE***

The level of specific IgE *Aspergillus* was measured in all patients using "HYTEC 288 (HYCOR Biomedical, Hannover, Germany)" according to the manufacturer’s manual. *Aspergillus*-specific IgE level greater than 0.35 (IU/ml) was considered to be positive.

***Total IgE and Aspergillus-specific IgG***

The enzyme-linked immunosorbent assay (ELISA) method was applied to measure the total IgE (Genesis ELISA Kit, Omega Diagnostic Group, UK) and *Aspergillus-*specific IgG (IBL ELISA Kit, Hamburg, Germany). *Aspergillus-*specific IgG levels were recorded for all asthmatic patients. According to manufacturer’s manual, the total IgE level of > 417 IU/ml and *Aspergillus-*specific IgG level of > 12 IU/ml were considered to be positive. Statistical analysis was performed in SPSS software (version 18).

## Results

Table 1 summarizes the baseline demographic characteristics of 200 patients with moderate (51.5%) to severe (48.5%) allergic bronchial asthma. Out of all participants, 111 (55.5%) subjects were female. The mean age of patients as well as the mean asthma duration were 45.8 (age range: 18-78) and 10.04±9.94 years, respectively. Furthermore, 100% and 57.5% (n=115) of asthmatic patients had a family history of atopy and asthma, respectively.

The mean levels of *Aspergillus***-**specific IgE, IgG, and total IgE were 1.5±6.01 (range: 0.1-61.3 IU/ml), 35.2±42.6 (range: 0-184 IU/ml), and 316.3±305.4 (range: 6-1300 IU/ml), respectively. The ranges of *Aspergillus***-**specific IgE and IgG levels in patients with severe asthma were 0.36-61.3 and 0-180 IU/ml, and in patients with moderate asthma were 0.42-19.89 and 0-184 IU/ml, respectively (Table 2). 

Table 3 shows that 27 (13.5%) and 22 (11.0%) patients had positive SPT and *Aspergillus-* specific IgE. In addition, *Aspergillus***-**specific IgE and SPT were positive in 16 (16.5%) and 15 (15.5%) patients with severe asthma, respectively. Total IgE levels of > 417 and > 1000 IU/ml were found in 65 (32.5%) and 6 (3.0%) patients of the study population, respectively. Furthermore, 86 (43.0%) asthmatic patients had *Aspergillus***-**specific IgG level of > 12 IU/ml; however, 46 (47.4%) patients with severe asthma had *Aspergillus***-***specific *IgG level of > 12 IU/ml.

**Table 1:** Demographic information of patients with asthma

**Type of asthma**		**Moderate n=103**		**Severe n=97**		**Total n=200**		
**Gender**		**Male**	**Female**	**Male**	**Female**	**Male**	**Female**	**Total **
Age	Range	(20-78)	(18-72)	(27-67)	(18-78)	(18-78)	(20-78)	(18-78)
	Mean	44	44.9	47.9	46.3	45.8	46.1	45.5
Duration of asthma	Year (Mean±SD)	6.6	8.5	13.1	12.3	9.4	10.5	10.04
History of atopy n (%)	Yes	51 (100)	52 (100)	38 (100)	59 (100)	89 (100)	111 (100%)	200 (100)
	No	-	-	-	-	-	-	-
Hospitalised n (%)	Yes	21 (41.2)	18 (34.6 )	28 (73.7 )	46 (78 )	49(55.1 )	64 (57.6 )	113 (56.5)
	No	30 (58.8)	34 (65.4)	10 (26.3)	13 (22)	40 (49)	47 (42.3)	87 (43.5)
History of asthma in family n (%)	Yes	24 (47.1)	28 (53.8)	27(71.1)	36 (61)	51 (57.3)	64 (57.6)	115 (57.5)
	No	27 (53)	24 (46.1)	11 (29)	23 (40)	38 (42.7)	47 (42.3)	85 (42.5)
Inhaled corticosteroid n (%)	Yes	50 (98)	50 (96.2)	34 (89.5)	55 (93.2)	84 (94.4)	105 (94.6)	189 (94.5)
	No	1 (2)	2 (3.8)	4 (10.5)	4 (6.8)	5 (5.6)	6 (5.4)	11 (5.5)
Oral corticosteroid n (%)	Yes	34 (66.7)	29 (55.8)	34 (89.5)	53 (89.8)	68 (76.4)	82 (73.9)	150 (75)
	No	17 (33.3)	23 (44.2)	4 (10.5)	6(10.2)	21 (23.6)	29 (26.1)	50 (25)

**Table 2:** The distribution of total IgE, specific IgE and IgG against *A.fumigatus *in asthmatic patients

**Type of ** **Asthma**	**Moderate**			**Severe**			**Total**		
**Gender**	**Male**	**Female**	**Total**	**Male**	**Female**	**Total**	**Male**	**Female**	**Total**
Total IgE (IU/ml)	(6-945)	(8-1047)	(6-1040)	(9-1300)	(7-1155)	(8-1300)	(6-1300)	(7-1155)	(6-1300)
	309.1 (±288.1)	236.2 (±275.5)	272.7 (±282.9)	423.8 (±367.9)	323.7 (±293.9)	362.9 (±326.8)	358.0 (±327.7)	282.7 (±287.5)	316.3 (±307.6)
*Aspergillus-specific * IgE (IU/ml)	(0.42-1.03)	(1.15-19.89)	(0.42-19.89)	(2.18-61.3)	(0.36-31.18)	(0.36-61.3)	(0.42-61.3)	(0.36-31.8)	(0.1-61.3)
	0.31 (±0.14)	1.1 (±3.5)	2.4 (±8.2)	4.1 (±11.6)	1.4 (±4.8)	0.7 (±2.5)	1.9 (±7.7)	1.2 (±4.2)	1.5 (±6.0)
*Aspergillus-specific * IgG (IU/ml)	(0-134)	(0-184)	(0-184)	(0-180)	(0-171)	(0-180)	(0-180)	(0-184)	(0-184)
	34.0 (±32.7)	32.5 (±41.5)	33.3 (±37.2)	49.6 (±55.9)	29.2 (±40.2)	37.2 (±47.8)	40.6 (±44.5)	30.8 (±40.7)	35.2 (±42.6)

**Table 3:** The frequency of positivity rate of patients with different type of asthma to *Aspergillus* skin test, total IgE, and specific IgE and IgG against *Aspergillus fumigatus*

**Variables **	**Type of asthma**	**Moderate ** **n= 103**	**Severe n=97**	**Male n=89**	**Female ** **n=111**	**Total** **n=200**
*Aspergillus* skin prick test	Positive	11 (10.7%)	16 (16.5%)	9 (10.1%)	18 (16.2%)	27 (13.5%)
Total IgE (IU/ml)	Range	(6-1040)	(8-1300)	(6-1300)	(7-1155)	(6-1300)
	(Mean± SD)	272.7±282.9	362.9±326.8	358.0±327.7	282.7±287.5	316.3±307.6
	> 417 n (%)	29 (28.2)	36 (37.1)	32 (36.0)	33 (39.7)	65 (32.5)
	< 1000 n (%)	103 (100%)	91 (93.8%)	85 (95.5%)	109 (98.2%)	194 (97%)
*Aspergillus-specific *IgE (IU/ml)	(Range)	(0.42-19.89)	(0.36-61.3)	(0.42-61.3)	(0.36-31.8)	(0.1-61.3)
	(Mean± SD)	2.4 (±8.2)	0.7 (±2.5)	1.9 (±7.7)	1.2 (±4.2)	1.5 (±6.0)
	Positive (>0.35IU/ml)	7 (6.8%)	15 (15.5%)	13 (14.6%)	9 (8.1%)	22 (11%)
*Aspergillus-specific *IgG (IU/ml)	(Range)	(0-184)	(0-180)	(0-180)	(0-184)	(0-184)
	(Mean± SD)	33.3 (±37.2)	37.2 (±47.8)	40.6 (±44.5)	30.8 (±40.7)	35.2 (±42.6)
	< 12 n (%)	40 (38.9%)	46 (47.4%)	32 (36.0%)	54 (48.6%)	86 (43.0%)

## Discussion

One of the important complications of fungal sensitization is the increased severity of asthma and mortality (13, 15). The prevalence of *Aspergillus* sensitization in patients with asthma varied from 15% to 48%, with a pooled prevalence of 28% (3, 16). It is estimated that approximately 6.5 million patients (33%) with severe asthma are affected with *Aspergillus* and fungal sensitisation in the world (17, 18). 

Unlike asthmatic patients with *Aspergillus* sensitization, patients with ABPA require a corticosteroid to control immune responses. Therefore, it is significantly important to consider the differences between these two patient groups (3). The diagnostic criteria to distinguish ABPA from *Aspergillus* sensitization include performing AST and measuring the levels of total IgE, *Aspergillus***-**specific IgG and/or *Aspergillus***-**specific IgE (3, 19). However, it is not reliable to simply diagnose ABPA or *Aspergillus* sensitization in asthmatic patients based on one of the mentioned parameters. 

In the present study, 13.5%, 32.5 %, and 11.0% of the patients with asthma had positive reaction to AST, total IgE, and specific IgE against *Aspergillus*, respectively. In a study conducted by Agarwal et al. (19), it was reported that 82.3% and 70.4% of asthmatic patients had IgE levels of > 417(IU/ml) and > 1000 (IU/ml), respectively. In addition, 47.3% of asthmatic patients showed positive reaction to AST (19). 

In several studies, the positive reaction rates to AST were reported as 21.5% (20), 27.6% (21), and 35.1% (22). Moreover, the levels of total IgE in the current study were low, compared to the results reported by the previous studies (19, 22). The obtained differences in positive rates may be due to varieties in genetic, nationality, race, and geographical and climatic conditions. 

In a study performed by Maurya et al. (22), 10.4% of asthmatic patients had positive reaction to *Aspergillus***-**specific IgG. However, in the present study, the level of *Aspergillus***-**specific IgG was 43.0% in patients with asthma. The determination of *Aspergillus***-**specific IgG level can be more helpful for the diagnosis of ABPA and allergic alveolitis or aspergilloma (23). However, the results of the present study on the evaluation of *Aspergillus***-**specific IgE levels were comparable with those of the previous studies (23). 

In the current study, the highest levels of total IgE and *Aspergillus-specific *IgG scattering were below 400 (IU/ml) and 60 IU/ml, respectively. Moreover, the highest concentration of *Aspergillus-specific *IgE was observed at levels 0 to 10 IU/ml. These findings are consistent with the results of studies conducted on airborne *Aspergillus* species frequency in Iran (24-26). 

According to the results of the present study, the positive rate of AST, total IgE, specific IgE, and IgG against *Aspergillus* were higher in severe asthma patients than in patients with moderate asthma. Several studies reported that patients with severe asthma may immunologically be more sensitive to one or more fungi (27, 28). Given the fact that sensitivity to *Aspergillus* increases the severity of asthma, the examination of patients with asthma is very important for the evaluation of their sensitivity to *Aspergillus* allergens. 

Denning et al. (27) showed the positive effect of antifungal therapy on the improvement of respiratory symptoms in patients with severe asthma who were sensitive to *Aspergillus*. Therefore, the exact diagnosis of fungal sensitization is also important in patients with severe asthma affected by ABPA (29).

This study is limited due to the lack of information on healthy controls. Therefore, no comparisons were made between asthmatic patients and healthy controls in terms of *Aspergillus-specific *IgG and IgE levels. In addition, the asthmatic patients had no follow-up visits; consequently, the reduction or increase of *Aspergillus-specific *IgG and IgE levels were not evaluated in the study group.

## Conclusion

In conclusion, the results of the present study are in line with those of other studies in that the reactivity to *Aspergillus* is considered as a remarkable phenomenon in asthmatic patients. It is also emphasised that the climatic condition may have an influence on the positivity rate of hypersensitivity against *Aspergillus*. 
